# Variability in bimanual wheelchair propulsion: consistency of two instrumented wheels during handrim wheelchair propulsion on a motor driven treadmill

**DOI:** 10.1186/1743-0003-10-9

**Published:** 2013-01-29

**Authors:** Riemer JK Vegter, Claudine J Lamoth, Sonja de Groot, Dirkjan HEJ Veeger, Lucas HV van der Woude

**Affiliations:** 1 Center for Human Movement Sciences, University Medical Center Groningen, University of Groningen, Groningen, The Netherlands; 2 Reade, Center for Rehabilitation& Rheumatology, Amsterdam, the Netherlands; 3 Faculty of Human Movement Sciences, Research Institute MOVE, Vrije Universiteit, Amsterdam, The Netherlands; 4 Faculty of Mechanical, Maritime and Materials Engineering, section Biomechatronics & Biorobotics, Delft University of Technology, Delft, Netherlands; 5 Center for Rehabilitation, University Medical Center Groningen, University of Groningen, Groningen, the Netherlands

**Keywords:** (MeSH), Wheelchairs, Rehabilitation, Biomechanics, Motor skills

## Abstract

**Background:**

Handrim wheelchair propulsion is a complex bimanual motor task. The bimanually applied forces on the rims determine the speed and direction of locomotion. Measurements of forces and torques on the handrim are important to study status and change of propulsion technique (and consequently mechanical strain) due to processes of learning, training or the wheelchair configuration. The purpose of this study was to compare the simultaneous outcomes of two different measurement-wheels attached to the different sides of the wheelchair, to determine measurement consistency within and between these wheels given the expected inter- and intra-limb variability as a consequence of motor control.

**Methods:**

Nine able-bodied subjects received a three-week low-intensity handrim wheelchair practice intervention. They then performed three four-minute trials of wheelchair propulsion in an instrumented hand rim wheelchair on a motor-driven treadmill at a fixed belt speed. The two measurement-wheels on each side of the wheelchair measured forces and torques of one of the two upper limbs, which simultaneously perform the push action over time. The resulting data were compared as direct output using cross-correlation on the torque around the wheel-axle. Calculated push characteristics such as power production and speed were compared using an intra-class correlation.

**Results:**

Measured torque around the wheel axle of the two measurement-wheels had a high average cross-correlation of 0.98 (std=0.01). Unilateral mean power output over a minute was found to have an intra-class correlation of 0.89 between the wheels. Although the difference over the pushes between left and right power output had a high variability, the mean difference between the measurement-wheels was low at 0.03 W (std=1.60). Other push characteristics showed even higher ICC’s (>0.9).

**Conclusions:**

A good agreement between both measurement-wheels was found at the level of the power output. This indicates a high comparability of the measurement-wheels for the different propulsion parameters. Data from both wheels seem suitable to be used together or interchangeably in experiments on motor control and wheelchair propulsion performance. A high variability in forces and timing between the left and right side were found during the execution of this bimanual task, reflecting the human motor control process.

## Background

Handrim wheelchair propulsion is the means of ambulation for a large group of people with a disability. However, handrim wheelchair propulsion is straining and (overuse) injuries to the upper extremities, e.g. shoulder pain or carpal tunnel syndrome, among wheelchair-dependent persons are common [1-3]. Therefore, a better understanding of wheelchair skill, physical capacity and the impact of wheelchair mechanics and fitting on performance are important [4-6]. Research over the past 30 years has led to a number of studies on the physiology and biomechanics of wheeled mobility [7,8]. Due to the complexity of instrumentation this research was primarily lab-based. Only more recently ambulant instrumentation for both physiological and biomechanical outcomes became available, which today even evolved into commercially available clinical tools [9,10].

Measurements of forces and torques on the handrim of a wheelchair are important to study change of propulsion technique due to learning, training or the effect of changes to the wheelchair. From a scientific point of view this provides a deeper understanding of the universal principles regarding the motor control of wheelchair propulsion, while from a clinical perspective it can help to better tailor the properties of a wheelchair to a patients needs’ and develop intervention protocols with respect to propulsion technique and strategy [11]. Over time, different studies have used different ways to instrument the wheels to gain insight in the forces and timing involved in wheelchair propulsion, varying from instrumented ergometers to specialized wheels [9,10,12-16]. These measurement systems have been used to describe unilaterally the cyclic nature of handrim propulsion analogous to gait analysis. For example, frequency of pushes, peak forces and torques and the wheel angle covered within a push have been used to describe the motor learning process of novel wheelchair users [17]. Besides propulsion technique these wheels are able to measure the power output of the wheelchair-user combination, making it possible to calculate mechanical efficiency when combined with cardio-respiratory measurements and energy calculations [18].

Most studies on propulsion technique measured this essentially bimanual propulsion task unilaterally and focused on the description of propulsion characteristics in dependence of a variety of different interventions. Yet, due to both internal control processes and external perturbations interlimb variation is expected [19]. Studying unilateral wheelchair propulsive mechanics provide biomechanical information about propulsion technique measures like peak forces and push time. However, wheelchair propulsion is a bimanual task, and studies in the area of bimanual motor control have shown that the limbs are not controlled independently, but are coupled to each other. This implies that principles of interlimb coordination cannot be derived from the study of single-limb movements [20].

Only few studies addressed bimanual upper limb consistency or the variability for that matter of motor performance in this task by using two instrumented wheels simultaneously [16,21,22]. Moreover, the provided data on reliability or validity of these measurement-wheels or related ergometer technology in literature are very scarce [10,23-25], let alone the comparability of different measurement-wheels.

**Figure 1:**
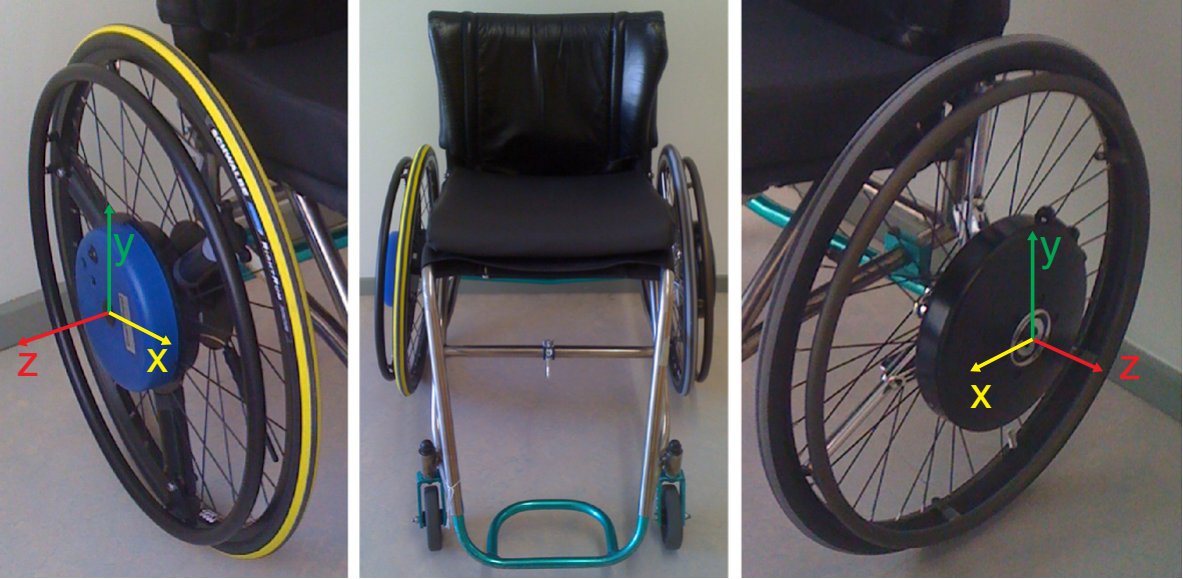
**Smartwheel**^**a **^**(left) and Optipush**^**b **^**(right).** Both wheels measure three-dimensional forces and torques on the handrim, combined with the angle under which the wheel is rotated. The local coordinate systems are defined as: x- and y-axis, orthogonal to each other in the plane of the handrim, z axis orthogonal to the plane of the handrim and pertruding from the wheel axle. The direction of positive torque is mirrored for the wheels so that they measure positive torque when pushing forward.

In order to evaluate the consistency of such measurement systems during steady-state wheelchair propulsion on a motor driven treadmill, the current study simultaneously assesses two commercially available instrumented wheelchair wheels: the Smartwheel^a^ and the Optipush^b^ (Figure 1). The Smartwheel uses instrumented beams to measure torques and forces, whereas the Optipush uses a commercial force-torque sensor at the center, which attaches to the rim through rigid beams. Both come with a clinical software package that can be used for guidance and evaluation of wheelchair adaptations and training programs in clinical or adapted sports practice. An important question is how these measurement-wheels compare to each other, and whether they consistently measure similar technique phenomena since they are based on different measurement approaches, yet are suggested to measure the same variables in the same range of accuracy. Thus from a clinical as well as a scientific perspective, it is important to know whether these wheels are interchangeable and whether we can compare the studies using these different wheels.

One way to compare the measurement-wheels is to fit them to both sides of the same wheelchair, during a steady-state propulsion task on a treadmill. Although both wheels examine the same performance of wheelchair propulsion, the motor control process involves a combined movement of the two upper limbs, which determines speed and direction. When comparing the pushes on both wheels, one faces the problem that human movement is intrinsically variable, both within and between individuals [26]. At present, there is a growing recognition that this variability (e.g. intra- and interlimb variability) is not simply the reflection of noise, but contains features that provide insight about normal learning and pathological processes [27-29]. Consequently for comparison of the wheels it is necessary to isolate the consistency of measurement from the variability inherent to the motor control process. Therefore a time dependent one-on-one comparison between pushes of the left and right arm are not expected to provide a suitable outcome measure for comparing the measurement-wheels against each other. Namely at this relatively small time-scale variability due to motor control and task variability, despite propelling at a constant speed on a motor driven treadmill, is to be expected. Indeed, one study using two Smartwheels specifically looked at the asymmetry of wheelchair propulsion and showed side-to-side differences when matching three pushes left and right [21].

Yet, the set task of straightforward steady-state propulsion on a level treadmill should be intrinsically stable over a larger time-scale and should lead to comparable mean outcome values for the left and right side, resulting in a constant mean power output (product of torque and angular velocity) over time. In the current study it is therefore assumed that systematic differences in unilateral mean power output between both wheels, when propelling at constant speed on a motor driven treadmill, should indicate differences in measurement systems rather then motor variability. For instance van der Woude et al. showed a high correlation (r=0.97) of left- and right-hand sprint power averaged over 30 seconds for 67 wheelchair athletes on a computer controlled wheelchair simulator [30]. Although steering on this ergometer is not critical like on a motor driven treadmill, this high interlimb consistency in power production still exists.

Traditionally power output on a motor-driven treadmill is determined through a separate drag test [31]. In the current study, the drag test, combined with the use of a pulley system is used to impose an additional drag force of known magnitude to the wheelchair-user combination on the treadmill [32]. The outcomes of the measurement wheels are compared with this other form of measuring power output.

Specifically we studied 1) if the two measurement-wheels (Optipush^b^, Smartwheel^a^) provided comparable time-averaged data for the left and right hand side during steady-state wheelchair propulsion on a motor-driven treadmill in a group of trained able-bodied subjects and 2) if the power output values for the measurement-wheels were comparable to the power output based on a separate drag test.

Answering these questions will enable researchers and practitioners to better interpret results from both measurement-wheels published in previous studies, and use both wheels in the same evaluation setup in the future. Furthermore it gives information on how earlier estimations of power output, using a drag test and a pulley system, compare to the outcomes of the measurement-wheels. Finally this study will help to further our understanding of details of bimanual variability in propulsion technique during steady-state handrim wheelchair propulsion.

## Methods

### Subjects

After having given written informed consent, 9 able-bodied subjects participated in the study. Criteria for inclusion were male, between 18–65 years, no prior experience in wheelchair propulsion, and absence of any medical contra-indications. To compare with earlier research in our laboratory only male subjects were selected. The study was performed according to the guidelines of the Ethics Committee of the Faculty of Human Movement Sciences, VU University Amsterdam (ECB 2011–46).

### Protocol

**Figure 2:**
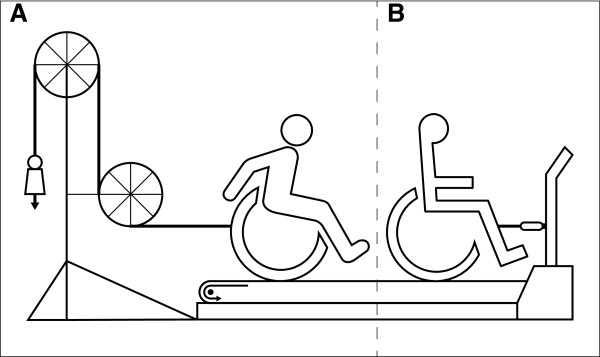
**Experimental setup. A**) To impose the desired power output a pulley-system attaches to the instrumented wheelchair on the treadmill. **B**) A dragtest is performed beforehand, to determine power output of the user-wheelchair combination.

Prior to our study, subjects practiced wheelchair propulsion in 9 practice trials over 3-weeks. Every trial comprised two 4-min exercise blocks at variable low-intensity levels of external power output. The first and the last trial were used as a pre- and post-test and were both extended with one 4-min exercise block. 2-Min rest was given between any two adjacent exercise blocks. Subjects received no specific instructions other than to stay on the treadmill using the handrims. The data for the current study were taken from the post-test that thus consisted of three four-minute blocks (T1, T2, T3) at 1.11 m/s and 0.18 W/kg. Figure 2 shows how the power was imposed by adding mass to a pulley system after having performed an individual drag test [31,32]. Experiments and practice sessions were all conducted on a level treadmill of 2.4 m length by 1.2 m width (Forcelink^c^) in the same experimental wheelchair (Double Performance^d^) with 24-inch measurement-wheels.

### Measurement-wheels

The regular rear 24 inch wheels of the standardized wheelchair were replaced with two instrumented wheels; on the left the OptiPush^b^ (6.0 kg, Max Mobility) and on the right the Smartwheel^a^ (4.9 kg, 3-Rivers). Both wheels measure 3-dimensional forces and torques applied to the handrim, combined with the angle under which the wheel is rotated. These variables were the only ones used in this experiment for data processing; further data processing and interpretation as done by the respective software packages was not included in the current study. Data were wirelessly transferred to a laptop at 200 Hz (Optipush) and 240 Hz (Smartwheel). Both wheels were synchronised by an electronic pulse at the start of each measurement.

### Data analysis

**Figure 3:**
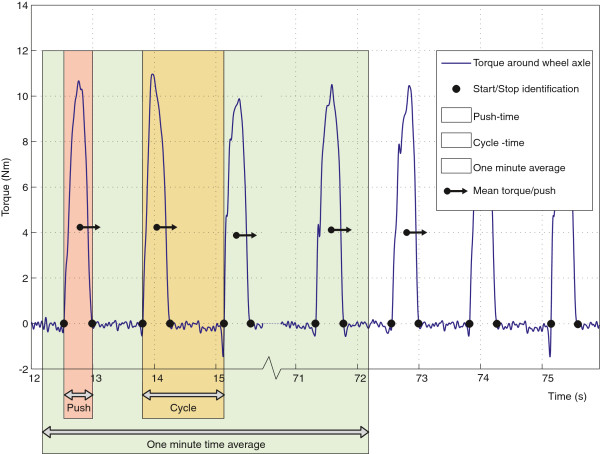
**Definitions of push variables.** Push identification, push-time, cycle-time, work per push, and mean torque. Variables were calculated per puss or over all full push cycles within one minute.

The rawest data from the instrumented wheels available to the researchers were further analysed using custom-written Matlab routines. Data of all three practice blocks including the rests in between were collected in one continuous measurement. To be sure of stable, steady-state propulsion, each last minute from the three 4-min exercise blocks (T1-T3) was used for the analysis. After data collection the Smartwheel output (240 hz) was downsampled to the frequency of the Optipush (200 hz) using a cubic spline. Per subject and exercise block, nine columns of data output were further used in the comparison between the measurement-wheels. These were the x, y and z components of force (N) and torque (Nm) as expressed by the wheels in their local coordinate systems (Figure 1), angle (rad), time (s) and sample number. First, individual pushes were defined as each period of continuous positive torque with a minimum of at least 1 Nm. Over the identified pushes biomechanical characteristics were calculated and later averaged over all pushes within the fourth minute of each practice block per subject. Calculated characteristics are defined in Table 1 and Figure 3.

**Table 1:** Propulsion variables

**Variable:**	**Description:**	**Equation:**
Push time (s)	Time from the start of positive torque to the stop of positive torque for an individual push.	t_end_(i) - t_start_(i)
Cycle time (s)	Time from the start of positive torque to the next start of positive torque.	t_end_(i) − t_end_(i − 1)
Contact angle (rad)	Angle at the end of a push minus the angle at the start.	Ø_end_(i) − Ø_start_(i)
Fpeak (N)	3d peak force during the push phase	Max_start : end_(Fx^2^ + Fy^2^ + Fz^2^)^0.5^
Mean Power/push (W)	The mean power during the push phase.	Mean_start : end_(Tz • *Δ*Ø)
Work/push (J)	The power integrated over the duration of the push.	∑_start : end_(Tz • *Δ*Ø)
Frequency (push/min)	Pushes per minute.	N_pushes_/*Δ*t
Mean power/minute (W)	The mean total (unilateral) power (Tz*Angular velocity) during a complete series of cycles in a minute multiplied by 2.	2 • (∑_start(i) : start(n)_(Tz • *Δ*Ø))

Abbrevations: t, time(s); _start_(i), start of the current push (sample); _end_(i), end of the current push (sample); Ø, angle (rad); Fx, Fy and Fz, force components (N); Tz, torque around wheel axle (Nm).

### Statistics

First, a cross-correlation was performed between the torque signals around the axle of each of the wheels for each subject, over the whole last minute of each practice block. We were specifically interested in correlation and the time lag between the two measurement-wheels. Possible differences in correlation and time lag were evaluated with repeated-measures Anova.

Secondly, the different biomechanical variables averaged over a minute were compared between the measurement-wheels with an intra-class correlation (ICC) over the different trials. A case 3 ICC was used to compare the degree of absolute agreement of the measurements that are the averages of the three independent 4-minute blocks with the two measurement-wheels as fixed judges [33]. A case 1 ICC was also performed over the three 4-minute blocks within each wheel to relate these within wheel outcomes to the between wheel outcomes. ICC values higher than 0.85 are considered good and measures below 0.7 as poor [34]. To further inspect the differences in power output Bland-Altman plots and limits of agreement were used [35].

Finally the total mean power output measured from the wheels was compared to that estimated by the drag test - pulley combination using an Anova for the different mean power outputs of the different measurement systems. Overall statistical significance was set at p<0.05.

## Results

The nine male subjects had a mean age of 25.9 years (std = 9.6), a mean body mass of 90.3 kg (std = 12.5) and a mean height of 1.90 m (std = 0.04). All subjects enrolled in the study after 8 sessions (1 first session of 12min and 7sessions of 8min) of low-intensity steady-state wheelchair exercise on the motor driven treadmill.

### Cross-correlation

Table 2 shows the cross-correlation between the torque signals around the wheel-axle (Figure 3) of both measurement-wheels for the three different practice blocks for each subject separately as well as the mean over n=9 subjects. For all three blocks we found a high cross correlation (respective means: 0.97, 0.97, 0.98) that did not change significantly (p=0.46). However, the lag between the two signals (see Table 2) for the three blocks did change significantly from a mean of 7.2 samples on T1 to 19.3 on T2 and 31.7 on T3 (p<0.001), indicating a shift in time of 0.06 ms between the signals obtained by both wheels between each practice trial.

**Table 2:** Cross correlation and the corresponding phase lag between the two torque signals around the wheel axis, for the different trials

	**T1**		**T2**		**T3**	
**Subjects**	**Cross correlation**	**lag**	**Cross correlation**	**lag**	**Cross correlation**	**lag**
1	0.97	8	0.98	21	0.97	33
2	0.98	4	0.97	17	0.98	31
3	0.98	7	0.98	17	0.98	28
4	0.96	7	0.97	20	0.98	33
5	0.96	11	0.97	23	0.97	32
6	0.98	8	0.97	17	0.98	32
7	0.98	4	0.98	17	0.98	29
8	0.97	8	0.97	20	0.97	32
9	0.96	8	0.96	22	0.97	35
**Mean:**	**0.97**	**7.2**	**0.97**	**19.3**	**0.98**	**31.7**
**Std:**	**0.01**	**2.2**	**0.01**	**2.4**	**0.01**	**2.2**

### Intra-class correlation

Mean power output between both wheels had a good intra-class correlation of 0.89 (Table 3). Within the different wheels the ICC for mean power output over the three 4-minute blocks was considerably higher, 0.97 and 0.98 for the Optipush and Smartwheel respectively. For the other biomechanical variables ICC’s between the wheels are high (>0.9), indicating good agreement between both wheels. The variables that took more calculation steps (Work/push, mean power output two-sided and speed) had lower ICC’s (Table 3).

**Table 3:** Means and standard deviation (between brackets) of propulsion characteristics for the different measurement-wheels (Optipush (Op) en Smartwheel (Sw)) over the three 4-minute blocks (n=9 AB subjects)

**Variable:**	**T1 Op**	**T1 Sw**	**T2 Op**	**T2 Sw**	**T3 Op**	**T3 Sw**	**ICC Betw.**	**ICC Op Within**	**ICC Sw Within**
**Push time (s)**	0.37 (0.05)	0.38 (0.05)	0.38 (0.06)	0.37 (0.06)	0.37 (0.06)	0.38 (0.07)	0.97	0.96	0.95
**Cycle time (s)**	1.24 (0.24)	1.29 (0.27)	1.29 (0.31)	1.24 (0.25)	1.27 (0.27)	1.28 (0.31)	1	0.94	0.95
**Contact angle (rad)**	1.36 (0.16)	1.37 (0.19)	1.38 (0.2)	1.33 (0.2)	1.34 (0.21)	1.37 (0.24)	0.97	0.96	0.95
**Fpeak (N)**	66.87 (14.75)	68.92 (16.72)	67.47 (16.34)	63.47 (16.25)	65.25 (19.72)	62.79 (17.3)	0.94	0.92	0.94
**Work/push (J)**	10.47 (1.76)	10.66 (2.15)	10.72 (2.34)	10.21 (1.88)	10.38 (2.78)	10.57 (2.59)	0.89	0.91	0.94
**Mean Power/push (W)**	27.73 (5.21)	27.8 (5.35)	27.67 (5.34)	27.82 (5.82)	27.79 (7.01)	27.84 (6.25)	0.9	0.93	0.96
**Frequency (push/min)**	50.69 (8.87)	49 (9.5)	49.55 (10.16)	50.33 (8.92)	49.27 (9.2)	49.47 (10.18)	1	0.94	0.95
**Mean power/ minute (W)**	16.4 (2.48)	16.05 (2.63)	16.11 (2.48)	16.28 (2.39)	16.08 (2.61)	16.31 (2.47)	0.89	0.97	0.98

The first ICC is between the wheels. Reported ICC’s in the last 2 columns are calculated between the mean per subject of the three blocks for each of the wheels.

### Bland altman plots

**Figure 4:**
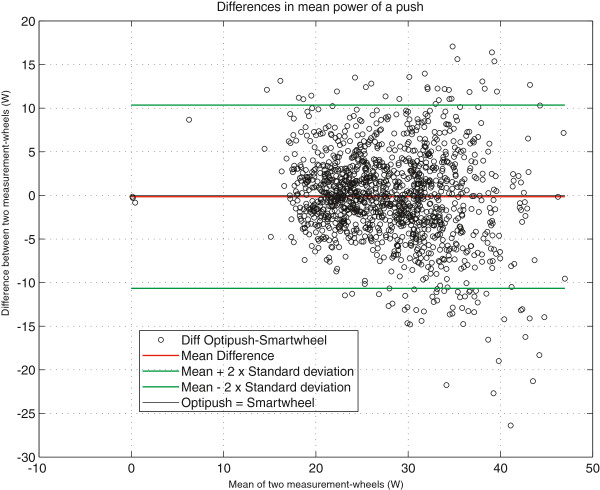
**Bland-Altman plot of all pushes, for all practice blocks of all subjects.** Each push of the Optipush^b^ is matched to the simultaneous one of the Smartwheel^a^. For each push the difference of the two is plotted against the mean of the two (black circles). It is clearly seen that they differ a lot on individual pushes, but on average (red line) there is no clear systematic difference between the wheels.

**Figure 5:**
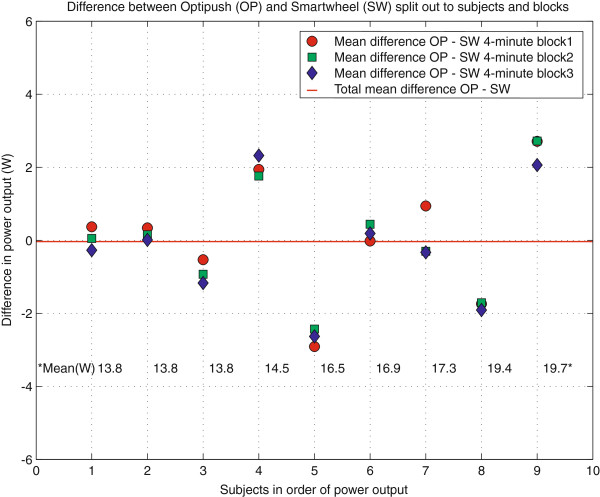
**Adjusted Bland Altman plot.** The differences between Optipush^b^ and Smartwheel^a^ are split out for each subject (x-axis) for each 4-minute block (symbols). Subjects are ranked according to their mean power output level.

The results on mean power output per push are shown in the Bland Altman plot in Figure 4. In this plot over n=9 subjects, each individual push of the Optipush has been matched to a time-synchronized push of the Smartwheel and the difference of these pushes is plotted against the mean of those two pushes. As expected, differences between left and right occurred, but the mean difference over the group and measurement period was very close to zero (−0.03 W). This low mean difference over the group and time, exemplifies that mean power left and right did not differ significantly. Further, Figure 5 shows the average power output over one minute for each subject (displayed on the x-axis) for each 4-minute block (displayed as different markers). The figure shows that for the individual subjects’ differences in power did occur as a consequence of the human motor control process and assumed detailed elements of task variation. Yet on group level (average difference over the group) no systematic differences were found (Mean difference −0.03 W).

### Measurement-wheels and drag test

Mean total power output was compared for both wheels and with an external criterion; the calculations of a drag test in combination with a pulley system shown in Table 4, column 3. The measurement-wheels did not significantly differ from each other (means 16.2 and 16.2 W, p=0.73), but measured a significantly higher power output than estimated from the drag test – pulley combination (mean 14.0 W, p<0.00).

**Table 4:** Power output (Pout) as determined by three different methods. There is a significant difference between the measurement-wheels and the drag test

**Subject**	**Handedness**	**Mean Pout x2 Op(Watt)**	**Mean Pout x2 Sw(Watt)**	**Pout dragtest (Watt)**
1	Right	18.5	20.3	15.8
2	Left	17.0	16.8	13.2
3	Right	13.9	13.7	13.7
4	Left	15.5	13.5	11.1
5	Right	13.4	14.3	11.6
6	Right	15.2	17.9	16.0
7	Right	17.4	17.3	15.9
8	Right	13.8	13.8	11.9
9	Right	21.0	18.5	16.5
**Mean:**		**16.2**	**16.2**	**14.0**

## Discussion

The aim of the present study was to compare two different measurement-wheels with supposedly the same data output under a real life dynamic, yet standardized submaximal wheeling condition. The results will help interpretation in future research regarding wheelchair propulsion with the use of these wheels, and will help to further investigate the intricate interlimb coupling during this bimanual task. The results showed good agreement between both wheels during steady-state propulsion on a treadmill. Both in time (cross-correlation) and in amplitude (intra-class correlation) a high correlation between the wheels was found (Tables 2 and 3). With regard to the power output both wheels showed comparable and consistent results.

### Cross correlation

The directly measured torque signals had a high cross-correlation, but over the different 4-minute blocks the time lag between the two signals became larger, from 0.04 s after 4 minutes to 0.16 s after 12 minutes. While the wheels were synchronized at the start a synchronous stop was not possible for the Optipush. The two internal clocks of both devices probably differed, yet in the current setup it was not possible to say in which way since a third source of known reliability was not available. Had a synchronous stop been possible we could have corrected for this phenomenon. Despite the small magnitude such options would be greatly appreciated in the future, especially if these measures are to be combined with other measurement systems like EMG or position registration.

### Intra-class correlation

Most push characteristics had an ICC higher than 0.9. The timing parameters push time, cycle time and frequency approach an ICC of 1.00 and so did the contact angle. These results all added to the conclusion that the provided signals by both wheels were highly comparable in the time and space profiles. The force-related parameters peak force, work per push and power had slightly lower ICC’s, but were still sufficiently high given that they measured different limb actions, which force profiles had to be produced individually and might also depend on hand dominance [36,37]. Since the data were collected within a larger framework of experiments it was decided not to change the sides of the different measurement-wheels, which could have shown differences due to hand dominance. Yet the studies that did look into the interlimb coupling and relationship between dominant and non-dominant hand in wheelchair propulsion did not yet show a clear effect of handedness [16,21]. Future experiments using both wheels could further investigate this possible confounder of the results.

### Bland altmann plots

**Figure 6:**
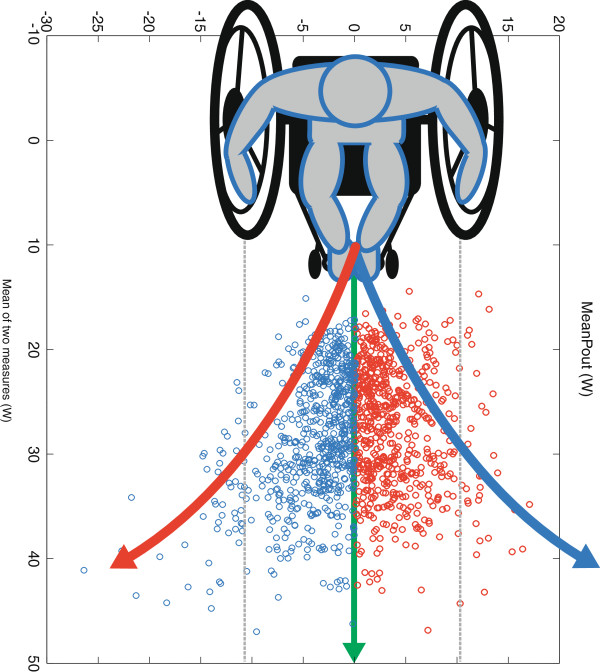
**Top view visualization of steering and propulsion.** This adapted version of Figure 4 shows the effect of the differences in mean power output of individual pushes with regard to steering. Blue circles are pushes where the Smartwheel measured more power leading to a change in the direction of the blue arrow. Vice versa for the Optipush the red circles lead to a change in the direction of the red arrow. Result is the green arrow, which was the overall outcome (staying on the treadmill).

The coupling of propulsion to steering in real life and on the motor driven treadmill seems to make wheelchair propulsion an intrinsic variable task requiring continuous coordination by the human motor system. Considerable left-right differences were found when comparing single pushes, showing variation in power output between the different sides, as was expected from a motor control perspective [19] The Bland Altman plot in Figure 4 is alternatively visualized in Figure 6. This figure illustrates how left-right differences influence direction and how eventually subjects manage to stay on the treadmill. Whether wheelchair propulsion is considered an asymmetrical act depends on the research interest. Clearly pushes left and right are not exactly the same and even differ considerably from time to time. As such, research fields like motor learning would greatly benefit from the use of two wheels to see how this variability changes because of a practice or feedback intervention. On the other hand over a larger time scale in a straightforward steady state submaximal propulsion task both limbs show very comparable propulsion performance allowing for generalization of findings from one side to the other. For instance seat height changes might be studied with just one measurement-wheel [38].

### Left-right power output

Power output is a complex output measure, using different components of a measurement-wheel, in this case torque multiplied by angular velocity averaged over time. Assuming a balanced, well-maintained and good quality wheelchair system on a stable and level treadmill, average power output at the left and right side should be identical over time during steady-state wheelchair propulsion on a motor driven treadmill. Although on group level the average difference in power output indeed was almost zero, individual subjects clearly showed differences between the mean power output left and right (Figure 5). Accordingly the ICC for mean power output was lower than for some of the other propulsion characteristics and remarkably lower than the ICC within the measurement-wheels for the three different trials. This means that as expected mean power output of the three different trials was more consistent within the wheels than between wheels.

The individual differences in power output left and right might have different causes. First, dependent on the weight distribution of the subject in the chair and the position fluctuations of the subject-wheelchair combination on the treadmill belt, rolling friction in both the rear wheels and the front castor wheels might be different left and right. More weight on the left or right front castor wheel will increase friction on that side, leading to a higher necessary power output on that side. Second, the weight distribution by the subject between both wheels also proportionally influences the power output; leaning over to one side makes the power output on that side necessarily larger. Third, the pulley system was positioned in line with the center of the treadmill, however subjects propel an approximately 0.75 m wide wheelchair on a 1.20 m wide treadmill, which allowed for movement toward either side of the treadmill. Propelling the chair more to the side will give a force component from the pulley system orthogonal to the wheels resulting in more power output on the outer side with respect to the pulley system. Also, the belt tension of the treadmill is somewhat different at the sides versus the center of the belt, which may also lead to slightly higher levels of rolling resistance when coasting left or right on the treadmill instead of in the middle.

Finally, the wheels under study might be of influence on the measured power output. The differences in mass and inertia of both wheels could potentially have influenced left-right power output. The suppliers did unfortunately not make inertial properties available, which would be greatly appreciated in the future development. Secondly the two wheels could measure torque and angle differently resulting in different power output. While the first three arguments are assumed to be distributed equally over all subjects, the argument of the measurement-wheels is a systematic difference. Although the other reasons might have masked a difference due to the measurement-wheels, the absence of a systematic difference in power output or any of the other outcomes in our view supports the conclusion that differences between subjects seem to be caused rather by their own propulsion behavior and geometrical characteristics than by the measurement systems used.

The agreement in power output as found in the present study is in line with earlier studies [30,39]. In different experimental setups these studies also found good agreement on power output for both sides. Important in this respect is also the operationalization of the term power output. As mentioned in the introduction Hurd et al. [21] averaged the power over only three push cycles while the other studies averaged power over more cycles. Therefore their finding of asymmetry in power output, averaged over just three consecutive pushes, seems to be in line with our finding of high variability in the left-right difference in power output (Figure 4).

### Measurement-wheels and dragtest

The wheels measured more power than estimated from the dragtest-pulley combination. In addition to the aforementioned consequence of task variation on power output other factors might have contributed to this difference. First the drag test was performed without speed changes, but at a constant speed. Secondly, additional losses when going to the front and back of the treadmill or left-right are not measured by the drag test, but are measured by the wheels. Thirdly, the friction on the front wheels is also dependent on weight distribution. During the drag test subjects were seated in a uniform position (sitting upright with the trunk; hands on the lap), while during propulsion they were free to move in their wheelchair (e.g. with trunk flexion/extension) possibly leading to more rolling friction and thus a higher power. The measurement-wheels seem to measure power in a more accurate way, because they are sensitive to change of torque and angular velocity, still the drag test is the only external comparison currently possible and is relatively cost effective and easy to use

## Conclusion

A good agreement between both measurement-wheels was found in this study. Data from both wheels seem consistent and suitable to be used together in experiments on wheelchair propulsion. Both wheels measure a higher mean total power output compared to the estimation of power output using a drag test. If a standardization of an experiment is done using a drag test this should be taken into account. Variability in the execution of wheelchair propulsion seems an essential part in the motor control of this bimanual task. Further research into the interlimb coupling during this bimanual task might use bilateral measurement-wheels to explain and understand the variability between and within the push cycles of both wheels.

## Endnotes

^a^ Three Rivers Holdings, Mesa, AZ, USA.

^b^ MAX Mobility, LLC, Antioch, TN, USA.

^c^ Forcelink b.v, Culemborg The Netherlands.

^d^ Double Performance BV, Gouda, The Netherlands.

## Abbreviations

Op: Optipush ^b^; Sw: Smartwheel ^a^; Pout: Power output; ICC: Intraclass Correlation; Anova: Analysis of Variance

## Competing interests

The authors declare that they have no competing interests.

## Authors’ contributions

RJKV was involved in the conception of the research project, the design, the execution, the analysis and the interpretation thereof and the writing of the manuscript. CJL and SG were involved in the design, analysis and interpretation of the data analysis and writing of the manuscript. HEJV contributed to the conception of the study, interpretation of the data analysis and revising the manuscript. LHVW participated in the conception and organization of the study, interpretation of the data analysis and revising of the manuscript. All authors read and approved the manuscript.

## References

[B1] Akbar M Balean G Brunner M Seyler TM Bruckner T Munzinger J Grieser T Gerner HJ Loew M Prevalence of rotator cuff tear in paraplegic patients compared with controls J Bone Joint Surg Am 2010 92 23 30 20048092 10.2106/JBJS.H.01373

[B2] Alm M Saraste H Norrbrink C Shoulder pain in persons with thoracic spinal cord injury: prevalence and characteristics J Rehabil Med 2008 40 277 283 10.2340/16501977-0173 18382823

[B3] van Drongelen S de Groot S Veeger HE Angenot EL Dallmeijer AJ Post MW van der Woude LH Upper extremity musculoskeletal pain during and after rehabilitation in wheelchair-using persons with a spinal cord injury Spinal Cord 2006 44 152 159 10.1038/sj.sc.3101826 16151450

[B4] Van Den Berg R De Groot S Swart KM Van Der Woude LH Physical capacity after 7 weeks of low-intensity wheelchair training Disabil Rehabil 2010 32 26 2244 2252 10.3109/09638288.2010.535688 21110694

[B5] Richter WM Kwarciak AM Guo L Turner JT Effects of single-variable biofeedback on wheelchair handrim biomechanics Arch Phys Med Rehabil 2011 92 572 577 10.1016/j.apmr.2010.11.001 21440701

[B6] Morrow MM Hurd WJ Kaufman KR An KN Shoulder demands in manual wheelchair users across a spectrum of activities J Electromyogr Kinesiol 2010 10.1016/j.jelekin.2009.02.001 PMC2794990 19269194

[B7] Vanlandewijck Y Theisen D Daly D Wheelchair propulsion biomechanics: implications for wheelchair sports Sports Med 2001 31 339 367 10.2165/00007256-200131050-00005 11347685

[B8] van der Woude LH de Groot S Janssen TW Manual wheelchairs: research and innovation in rehabilitation, sports, daily life and health Med Eng Phys 2006 28 905 915 10.1016/j.medengphy.2005.12.001 16504565

[B9] Cooper RA SMARTWheel: from concept to clinical practice Prosthet Orthot Int 2009 33 198 209 10.1080/03093640903082126 19658010 PMC2739657

[B10] Kwarciak AM Rodriguez RE Sarkar N Richter WM Guo A Validation of a biofeedback system for wheelchair propulsion training Rehabilitation Research and Practice 2011 2011 590780 22110977 10.1155/2011/590780 PMC3196933

[B11] De Groot S Vegter RJK Vuijk C van Dijk FW van der Woude L Gelderblom GJ WHEEL-i: the development of a wheelchair propulsion lab for rehabilitation and sports Everyday Technology for Independence and Care 2011 29 Amsterdam: IOS Press

[B12] Richter WM Rodriguez R Woods KR Karpinski AP Axelson PW Reduced finger and wrist flexor activity during propulsion with a new flexible handrim Arch Phys Med Rehabil 2006 87 1643 1647 10.1016/j.apmr.2006.09.009 17141646

[B13] van Drongelen S van der Woude LH Janssen TW Angenot EL Chadwick EK Veeger DH Glenohumeral contact forces and muscle forces evaluated in wheelchair-related activities of daily living in able-bodied subjects versus subjects with paraplegia and tetraplegia Arch Phys Med Rehabil 2005 86 1434 1440 10.1016/j.apmr.2005.03.014 16003677

[B14] Desroches G Dumas R Pradon D Vaslin P Lepoutre F-X ChËze L Upper limb joint dynamics during manual wheelchair propulsion Clin Biomech 2010 25 299 306 10.1016/j.clinbiomech.2009.12.011 20106573

[B15] Limroongreungrat W Wang YT Chang L-s Geil MD Johnson JT An instrumented wheel system for measuring 3-D pushrim kinetics during racing wheelchair propulsion Res Sports Med: An International Journal 2009 17 182 194 10.1080/15438620903120637 19731178

[B16] De Groot S Veeger DH Hollander AP Van der Woude LH Wheelchair propulsion technique and mechanical efficiency after 3 wk of practice Med Sci Sports Exerc 2002 34 756 766 10.1097/00005768-200205000-00005 11984291

[B17] Kwarciak AM Sisto SA Yarossi M Price R Komaroff E Boninger ML Redefining the manual wheelchair stroke cycle: identification and impact of nonpropulsive pushrim contact Arch Phys Med Rehabil 2009 90 20 26 10.1016/j.apmr.2008.07.013 19154825

[B18] Lenton JP van der Woude LHV Fowler NE Nicholson G Tolfrey K Goosey-Tolfrey VL Hand-rim forces and gross mechanical efficiency at various frequencies of wheelchair propulsion Int J Sports Med 2013 34 2 158 22918717 10.1055/s-0032-1311650

[B19] Stergiou N Decker LM Human movement variability, nonlinear dynamics, and pathology: Is there a connection? Hum Mov Sci 2011 30 869 888 10.1016/j.humov.2011.06.002 21802756 PMC3183280

[B20] Swinnen SP Intermanual coordination: from behavioural principles to neural-network interactions Nat Rev Neurosci 2002 3 348 359 10.1038/nrn807 11988774

[B21] Hurd WJ Morrow MM Kaufman KR An KN Biomechanic evaluation of upper-extremity symmetry during manual wheelchair propulsion over varied terrain Arch Phys Med Rehabil 2008 89 1996 2002 10.1016/j.apmr.2008.03.020 18929029 PMC3899826

[B22] Boninger ML Souza AL Cooper RA Fitzgerald SG Koontz AM Fay BT Propulsion patterns and pushrim biomechanics in manual wheelchair propulsion Arch Phys Med Rehabil 2002 83 718 723 10.1053/apmr.2002.32455 11994814

[B23] Asato KT Cooper RA Robertson RN Ster JF SMART/sup Wheels/: development and testing of a system for measuring manual wheelchair propulsion dynamics Biomedical Engineering, IEEE Transactions on 1993 40 1320 1324 10.1109/10.250587 8125507

[B24] Cooper RA Boninger ML VanSickle DP Robertson RN Shimada SD Uncertainty analysis for wheelchair propulsion dynamics Rehabilitation Engineering, IEEE Transactions on 1997 5 130 139 10.1109/86.593279 9184899

[B25] DiGiovine CP Cooper RA DiGiovine MM Boninger ML Robertson RN Frequency analysis of kinematics of racing wheelchair propulsion IEEE Trans Rehabil Eng 2000 8 385 393 10.1109/86.867880 11001518

[B26] Newell K Corcos D Variability and motor control 1993 Champaign: Human Kinetics

[B27] Lamoth CJ Daffertshofer A Huys R Beek PJ Steady and transient coordination structures of walking and running Hum Mov Sci 2009 28 371 386 10.1016/j.humov.2008.10.001 19027972

[B28] Sparrow WA Lay BS O'Dwyer NJ Metabolic and attentional energy costs of interlimb coordination J Mot Behav 2007 39 259 275 10.3200/JMBR.39.4.259-275 17664169

[B29] Daffertshofer A Lamoth CJC Meijer OG Beek PJ PCA in studying coordination and variability: a tutorial Clin Biomech 2004 19 415 428 10.1016/j.clinbiomech.2004.01.005 15109763

[B30] van der Woude LH Bakker WH Elkhuizen JW Veeger HE Gwinn T Propulsion technique and anaerobic work capacity in elite wheelchair athletes: cross-sectional analysis Am J Phys Med Rehabil 1998 77 222 234 10.1097/00002060-199805000-00007 9635557

[B31] van der Woude LH de Groot G Hollander AP van Ingen Schenau GJ Rozendal RH Wheelchair ergonomics and physiological testing of prototypes Ergonomics 1986 29 1561 1573 10.1080/00140138608967269 3102225

[B32] Veeger D van der Woude LH Rozendal RH The effect of rear wheel camber in manual wheelchair propulsion J Rehabil Res Dev 1989 26 37 46 2724151

[B33] Shrout PE Fleiss JL Intraclass correlations: uses in assessing rater reliability Psychol Bull 1979 86 420 428 18839484 10.1037//0033-2909.86.2.420

[B34] Safrit M Wood T Introduction to measurement in physical education and exercise science 1995 3 Maryland Heights: Mosby

[B35] Bland JM Altman DG Statistical methods for assessing agreement between two methods of clinical measurement Lancet 1986 1 307 310 2868172

[B36] de Poel HJ Peper CE Beek PJ Handedness-related asymmetry in coupling strength in bimanual coordination: furthering theory and evidence Acta Psychol 2007 124 209 237 10.1016/j.actpsy.2006.03.003 16777042

[B37] Sainburg RL Evidence for a dynamic-dominance hypothesis of handedness Experimental brain research Experimentelle Hirnforschung Experimentation cerebrale 2002 142 241 258 10.1007/s00221-001-0913-8 11807578 PMC10710695

[B38] Kotajarvi BR Sabick MB An KN Zhao KD Kaufman KR Basford JR The effect of seat position on wheelchair propulsion biomechanics J Rehabil Res Dev 2004 41 403 414 10.1682/JRRD.2003.01.0008 15543458

[B39] Veeger HE van der Woude LH Rozendal RH A computerized wheelchair ergometer. Results of a comparison study Scand J Rehabil Med 1992 24 17 23 1604258

